# Pedicle morphology using CT-based navigation system in adolescent idiopathic scoliosis

**DOI:** 10.1186/1748-7161-8-S1-O27

**Published:** 2013-06-03

**Authors:** S Kuraishi, J Takahashi, H Hirabayashi, N Ogihara, K Mukaiyama, M Shimizu, H Kato

**Affiliations:** 1 Departments of Orthopaedic Surgery, Shinshu University, Matsumoto, Japan

## Background

Pedicle diameter in AIS patients was narrower on the concave side of the scoliotic curve. Many researchers have measured pedicle diameter and length of AIS patients by using standard CT, or magnetic resonance imaging (MRI), but only few have used three-dimensional (3D) imaging, especially CT-based navigation.

## Aim

The purpose of this study was to use multidimensional analysis with a computed tomography (CT)–based navigation system to measure the outer cortical diameter, and the maximum screw trajectory length, of the pedicle of the thoracic and lumbar regions of the spine in adolescent idiopathic scoliosis (AIS) patients. Another objective was to identify pedicles that require cautious insertion of screws.

## Methods

Fifteen patients with right-side thoracic AIS, who underwent pedicle screw fixation, were studied. CT-based navigation system was used to measure the pedicle diameter, defined as the widest outer cortical diameter at the narrowest part of the pedicle. Moreover, the maximum pedicle screw trajectory length was measured as the distance between the posterior cortical entry point of the pedicle screw, and the anterior vertebral cortex in line with the axis of the pedicle between T1 and L5. In addition, the values of each parameter taken using the CT navigation system, and the standard axial CT, were compared.

## Results

Pedicles on the concave side of the main thoracic curve apex and proximal thoracic curve tended to have the narrowest diameters. The mean length, of the longest screw that could be fixed, was longer on the right side, except for T8 and T9. Our data showed screw size feasibility as follows: 25 mm or 30 mm screws were feasible from T1 to T5; 30 mm or 35 mm screws, from T6 to T12; and 35 mm or 40 mm screws, from L1 to L5. Pedicle diameter measured by the CT navigation system was larger than that measured by standard axial CT. Left-side pedicle length measured by the CT navigation system was less than that measured by standard axial CT.

## Conclusion

Pedicle diameter in patients with AIS is narrower on the concave side of the scoliotic curve. Therefore, caution should be exercised during screw insertion on the concave side.
